# c-Src regulates δ-secretase activation and truncated Tau production by phosphorylating the E3 ligase Traf6

**DOI:** 10.1016/j.jbc.2023.105462

**Published:** 2023-11-15

**Authors:** Yanli Jiang, Longfei Li, Ruozhen Wu, Liulin Wu, Bin Zhang, Jian-Zhi Wang, Rong Liu, Fei Liu, Jing Wang, Xiaochuan Wang

**Affiliations:** 1 Department of Pathophysiology, School of Basic Medicine, Key Laboratory of Education Ministry/Hubei Province of China for Neurological Disorders, Tongji Medical College, Huazhong University of Science and Technology, Wuhan, China; 2 Co-innovation Center of Neuroregeneration, Nantong University, Nantong, Jiangsu, China; 3 Department of Neurochemistry, Inge Grundke-Iqbal Research Floor, New York State Institute for Basic Research in Developmental Disabilities, Staten Island, New York, USA; 4 Department of Immunology School of Basic Medicine, Tongji Medical College, Huazhong University of Science and Technology, Wuhan, China; 5 Shenzhen Huazhong University of Science and Technology Research Institute, Shenzhen, China

**Keywords:** Alzheimer’s disease, Tau pathology, truncation, Src, Traf6, AEP

## Abstract

The accumulation of abnormal Tau protein is a common feature of various neurodegenerative diseases. Truncated Tau, resulting from cleavage by asparaginyl endopeptidase (AEP, δ-secretase), promotes its own phosphorylation and aggregation. Our study focused on understanding the regulatory mechanisms of AEP activation and its interaction with other proteins. We discovered that c-Src plays a critical role in mediating the activation and polyubiquitination of AEP in response to epidermal growth factor stimulation. In addition, we investigated the involvement of tumor necrosis factor receptor–associated factor 6 (Traf6), an E3 ligase, in the regulation of AEP levels and its interaction with c-Src. Knockdown of Traf6 effectively inhibited c-Src-induced AEP activation. To gain further insights into the molecular mechanisms, we employed mass spectrometry to identify the specific tyrosine residues of Traf6 that are phosphorylated by c-Src. By mutating these phosphorylation sites to phenylalanine, we disrupted Traf6-mediated polyubiquitination and subsequently observed the inactivation of AEP. This finding suggests that the phosphorylation of Traf6 by c-Src is crucial for AEP activation. Pharmacological inhibition of c-Src reduced the phosphorylation of Traf6 and inhibited AEP activation in neurons derived from human-induced pluripotent stem cells. Conditional knockout of Traf6 in neurons prevented c-Src-induced AEP activation and subsequent Tau truncation *in vivo*. Moreover, phosphorylation of Traf6 is highly correlated with AEP activation, Tau368 and pathological Tau (AT8) in Alzheimer's disease brain. Overall, our study elucidates the role of c-Src in regulating AEP-cleaved Tau through phosphorylating Traf6. Targeting the c-Src–Traf6 pathway may hold potential for the treatment of Alzheimer's disease and other tauopathies.

Alzheimer’s disease (AD) is the most prevalent form of dementia and is marked by extracellular amyloid-β plaques and intracellular neurofibrillary tangles composed of hyperphosphorylated Tau (1). Tau undergoes various post-translational modifications (PTMs), such as phosphorylation, acetylation, sumoylation, ubiquitylation, and truncation (2). These PTMs may play a role in different stages of Tau aggregation (3). Among these PTMs, the truncation of Tau is linked to Tau pathogenesis in AD (4, 5). Truncated Tau is more likely to be phosphorylated and forms aggregates (6). Among the proteolytic enzymes that cleave Tau, asparaginyl endopeptidase (AEP), also known as δ-secretase, has been implicated in many neurodegenerative diseases (4, 7). AEP-cleaved Tau colocalized with paired helical filaments and contributes to the neurofibrillary pathology in AD (8). AEP is reported to be a lysosomal enzyme that is activated in an acidic environment by autoproteolysis to produce active mature AEP (mAEP) from its inactive zymogen pro-AEP (7). Its activity is regulated by PTMs, such as its phosphorylation by the serine/threonine kinase AKT or serine/threonine-protein kinase 2 (7). Moreover, tumor necrosis factor receptor–associated factor 6 (Traf6) is an E3 ligase, which can mediate the K63-linked polyubiquitination of AEP and recruit heat shock protein 90α (HSP 90α) to enhance AEP stability (9). Besides its lysosome localization, AEP is also found in the nucleus and cytosol (9).

As an enzyme, Traf6 has been reported to interact with Src family kinases, such as c-Src and Fyn, upon lipopolysaccharide stimulation (10). Both c-Src and Fyn are enriched in neurons and regulate Tau phosphorylation by inhibiting the activity of protein phosphatase 2A (PP2A), a major phosphatase that dephosphorylates Tau (11, 12). Fyn has been extensively studied in Tau pathogenesis and shown to regulate Tau aggregation *in vivo* (13). However, the roles of c-Src in the etiology of Tau pathology are poorly understood. Here, we explore whether c-Src regulates AEP activation and Tau truncation by modulating Traf6 activity in neurons.

We discovered that c-Src influences AEP activity by phosphorylating Traf6, which facilitates AEP-induced Tau truncation. Either pharmacologic inhibition of c-Src in neurons derived from human-induced pluripotent stem cells (hiPSCs) or genetic depletion of Traf6 in c-Src Y535F-overexpressing mice reduced mAEP levels and Tau truncation. c-Src regulates Tau phosphorylation by inhibiting PP2A activity; our study reveals that c-Src regulates AEP-cleaved Tau truncation by phosphorylating Traf6.

## Results

### c-Src mediates epidermal growth factor–induced AEP activation and polyubiquitination

**Figure 1:**
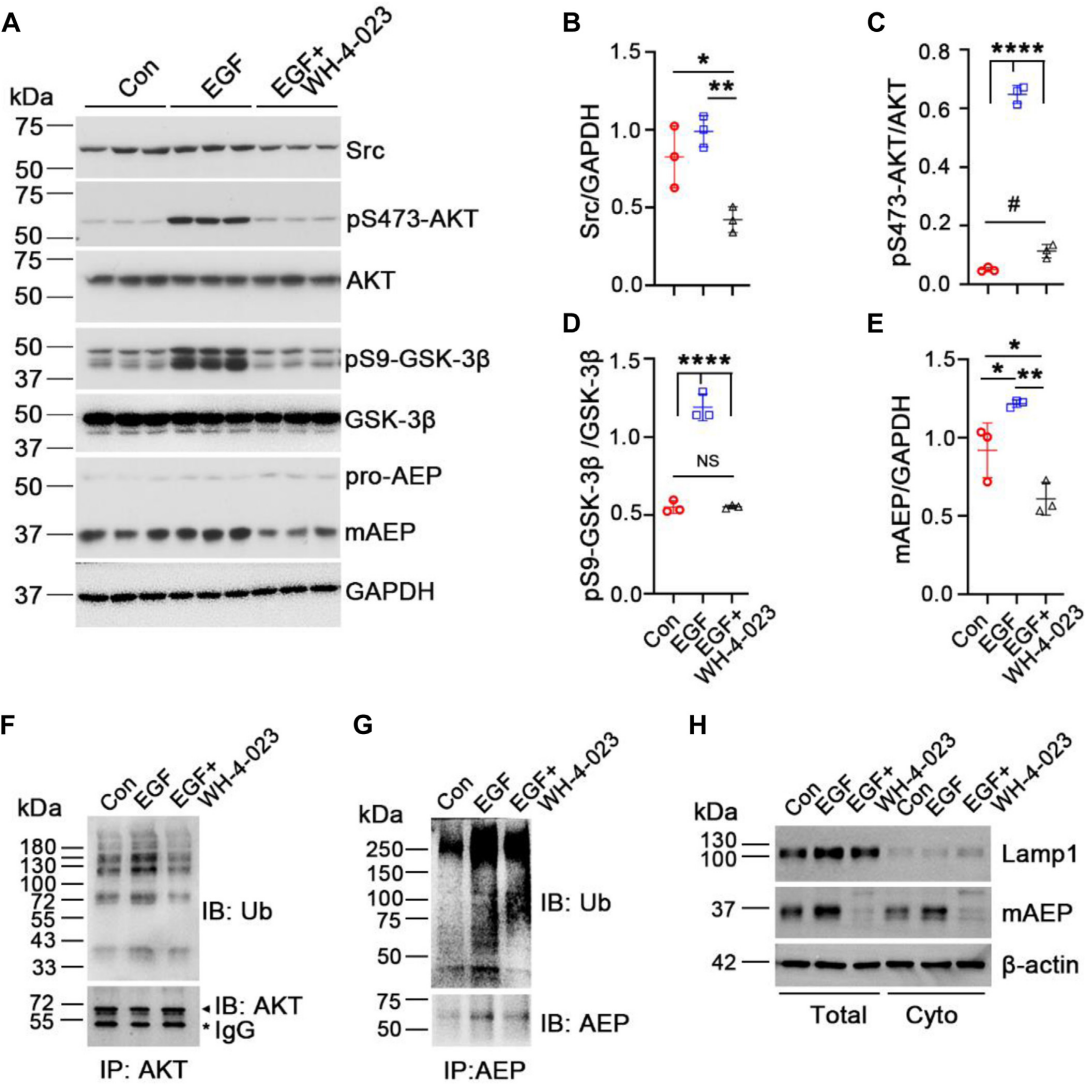
**c-Src mediates AEP activation in HEK-293T cells after EGF treatment.***A*, protein levels of c-Src, pS473-AKT, AKT, pS9-GSK-3β, GSK-3β, mAEP, and GAPDH were detected by Western blotting after EGF treatment with or without WH-4-023 treatment for 30 min. *B*, quantification of c-Src/GAPDH, (*C*) pS473-AKT/AKT, (*D*) pS9-GSK-3β/GSK-3β, and (*E*) mAEP/GAPDH. *F* and *G*, immunoblotting of the ubiquitination of immunoprecipitated AKT and AEP from cell lysates after EGF treatment with or without WH-4-023. ∗ indicates IgG heavy chain, and the *arrowhead* indicates AKT. *H*, cytosolic fraction isolated from total-cell lysate after EGF treatment with or without WH-4-023. AEP and β-actin were detected by Western blotting. Lysosome removal was confirmed by immunoblotting with the lysosome marker Lamp1. Data are presented as the mean ± SD. n = 3 for each group in *A*. Representative blots showed in (*F*–*H*) were from three repeated experiments with same results. ∗*p* < 0.05, ∗∗*p* < 0.01, ∗∗∗∗*p* < 0.0001, and # *p* < 0.05, NS indicates nonsignificant. AEP, asparaginyl endopeptidase; EGF, epidermal growth factor; GSK-3β, glycogen synthase kinase-3β; HEK-293T, human embryonic kidney 293T cell line; IgG, immunoglobulin G; mAEP, mature AEP.

To study whether c-Src regulates AEP activation and how c-Src regulates AEP activation, we treated starved human embryonic kidney 293T (HEK-293T) cells with epidermal growth factor (EGF). EGF binds its receptor and activates downstream signals, including the c-Src and AKT–glycogen synthase kinase-3β (GSK-3β) pathways (14, 15, 16). In addition, AKT activation requires c-Src activation (16, 17). Because the pY416-Src antibody recognizes Src family kinases and is not specific for c-Src, we measured AKT/GSK-3β phosphorylation as an indicator of c-Src activity. We observed that EGF treatment increased the phosphorylation of AKT at S473 and GSK-3β at S9, whereas a Src inhibitor (WH-4-023) reduced c-Src protein levels and suppressed the EGF-induced AKT–GSK-3β pathway (Fig. 1, *A*–*D*). In parallel to the inhibition of c-Src, mAEP levels were elevated after EGF treatment, and the Src inhibitor blocked the EGF-induced increase in mAEP (Fig. 1, *A* and *E*). These data suggest that c-Src mediates EGF-induced AEP activation.

Traf6 mediates K63-linked polyubiquitination and promotes AEP stability by recruiting HSP90α (9). Since c-Src interacts with Traf6 and phosphorylates Traf6 (10), we proposed that c-Src may regulate AEP activation through Traf6. Therefore, we tested whether EGF treatment promoted the polyubiquitination of AEP or other substrates of Traf6, such as AKT (as a positive control). After EGF treatment with or without WH-4-023, we detected the ubiquitination levels of AKT and AEP and found that inhibition of Src suppressed EGF-induced ubiquitination of AKT and AEP (Fig. 1, *F* and *G*). Because AEP is mainly localized to lysosomes and endosomes, we wondered whether c-Src-mediated AEP activation occurred in the cytosol. We removed lysosomes from whole-cell lysates, as indicated by the loss of the lysosome marker Lamp1 in Western blots and found that EGF treatment induced an increase in the mAEP level in the cytosol, which was suppressed by a Src inhibitor (Fig. 1*H*). This is consistent with other group’s findings that AEP was found in cytosol (9). Altogether, these findings suggest that c-Src mediates EGF-induced AEP ubiquitination and activation, probably through Traf6.

### c-Src regulates AEP activation by phosphorylating Traf6

**Figure 2:**
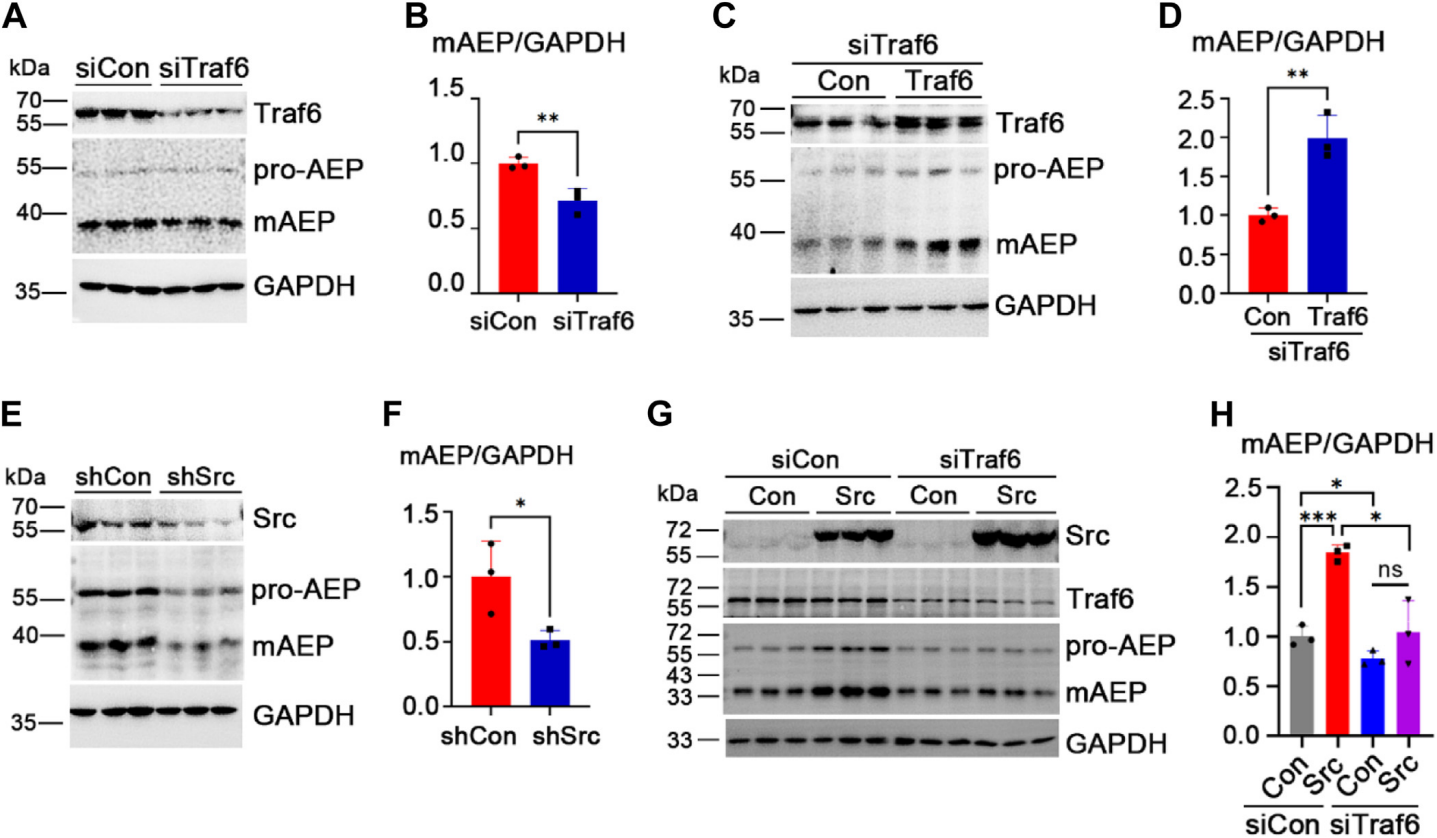
**c-Src regulates AEP activation through Traf6.***A*, Western blot showing Traf6 and AEP levels in HEK-293T cells with Traf6 knockdown. *B*, quantification of mAEP/GAPDH in (*A*). *C*, Western blot showing Traf6 and AEP levels in HEK-293T cells with Traf6 overexpression after Traf6 knockdown. *D*, quantification of mAEP/GAPDH in (*C*). *E*, Western blot showing c-Src, AEP, and GAPDH levels in HEK-293T cells treated with shCon and shRNA-Src. *F*, quantification of mAEP/GAPDH in (*E*). *G*, Western blot showing c-Src (FLAG), AEP, Traf6, and GAPDH levels in HEK-293T cells overexpressing c-Src with or without Traf6 knockdown by siRNA. *H*, quantification of mAEP/GAPDH in (*G*). Data are presented as the mean ± SD, n = 3 for each group. ∗*p* < 0.05, ∗∗*p* < 0.01, ∗∗∗*p* < 0.001, ∗∗∗∗*p* < 0.0001, ns indicates nonsignificant. AEP, asparaginyl endopeptidase; HEK-293T, human embryonic kidney 293T cell line; mAEP, mature AEP; Traf6, tumor necrosis factor receptor–associated factor 6.

Traf6 positively regulates pro-AEP levels, and mAEP is processed from pro-AEP (9, 18). Therefore, we hypothesized that Traf6 also regulates mAEP levels. To determine whether c-Src regulates AEP activation through Traf6, we first examined the effect of Traf6 on mAEP levels by knocking down Traf6 in HEK-293T cells using siRNA targeting the Traf6 3′UTR. mAEP levels were significantly decreased after Traf6 knockdown, as measured by Western blotting (Fig. 2, *A* and *B*). In contrast, overexpression of Traf6 significantly increased mAEP levels in HEK-293T cells transfected with siTraf6 compared with the control (Fig. 2, *C* and *D*). Because WH-4-023 is a dual inhibitor of Lck/Src, not a specific inhibitor of Src, we knocked down or overexpressed c-Src in HEK-293T cells to confirm the effect of c-Src on mAEP levels and found that c-Src-shRNA reduced mAEP levels, whereas overexpression of c-Src increased mAEP levels, suggesting that c-Src positively regulates mAEP levels (Fig. 2, *E*–*H*). In contrast, knockdown of Traf6 prevented the c-Src-induced increase in mAEP (Fig. 2, *G* and *H*). Altogether, our results suggest that c-Src regulates mAEP levels through Traf6.

**Figure 3:**
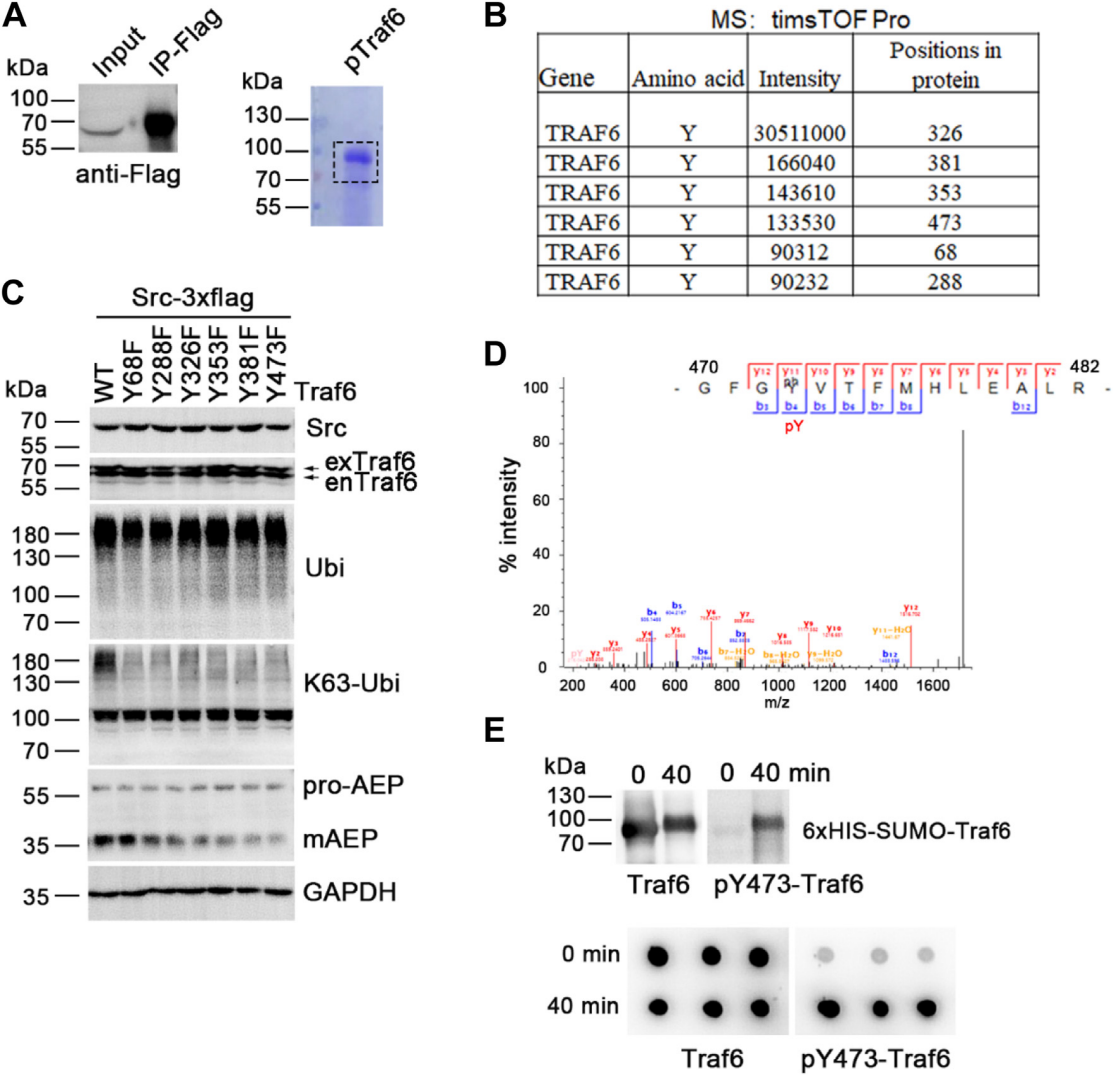
**c-Src regulates AEP activation by phosphorylating Traf6.***A*, FLAG-c-Src was overexpressed in HEK-293T cells and pulled down from the cell lysate. The presence of c-Src in the total-cell lysate and pull-down fraction was detected using an anti-FLAG antibody (*left*). *In vitro*, c-Src bound to anti-FLAG magnetic beads phosphorylates recombinant Traf6 for 40 min. Traf6 was loaded on SDS-PAGE gels and stained with Coomassie brilliant blue R-250 (*right*). The band consisting of Traf6 within the square was excised and subjected to mass spectrometry (MS) to detect the phosphorylation sites in Traf6. *B*, identification of the sites in Traf6 that are phosphorylated by c-Src using MS. *C*, mutations (Y68F, Y288F, Y326F, Y353F, Y381F, and Y473F) in Traf6 blocked K63-linked polyubiquitination in cells. The protein levels of K63-linked polyubiquitin, ubiquitin, c-Src, Traf6, and AEP were detected by blotting with the respective antibodies. GAPDH was used as an internal control. The *upper arrow* indicates exogenous overexpressed Traf6 with a 3×FLAG tag, whereas *lower arrow* indicates endogenous Traf6. *D*, spectra of pY in peptide 470-GFGYVTFMHLEALR-483 identified by MS. *E*, Western blotting and dot blotting of Traf6 and phosphorylated Traf6 probed by Traf6-pY473 and Traf6 polyclonal antibodies after a 40 min kinase reaction *in vitro*. AEP, asparaginyl endopeptidase; HEK-293T, human embryonic kidney 293T cell line; Traf6, tumor necrosis factor receptor–associated factor 6.

As a kinase, c-Src probably regulates Traf6 activity by phosphorylating it. To determine the mechanism by which c-Src regulates Traf6, we overexpressed c-Src in HEK-293T cells, immunoprecipitated it, and performed a kinase reaction with recombinant Traf6 *in vitro* (Fig. 3*A*). After reaction for 40 min, the sample was loaded for SDS-PAGE and Coomassie brilliant blue staining (Fig. 3*A*). The band consisting of Traf6 was cut out and processed for mass spectrometry (MS) identification. The protein sequence coverage of Traf6 was 76.8% in MS, and six tyrosine phosphorylation sites in Traf6 were identified (Figs. 3*B* and S1). To confirm whether the phosphorylation of Traf6 by c-Src regulates AEP activation, we coexpressed c-Src with Traf6 or nonphosphorylated mutants of Traf6 (six tyrosine phosphorylation sites were mutated to phenylalanine separately) in HEK-293T cells for 48 h. The Western blot results showed that the total ubiquitination level was not changed when the blot was probed with an ubiquitin antibody, which can recognize all forms of ubiquitination (Fig. 3*C*). As Traf6 is responsible for K63-linked polyubiquitination, we noticed that expression of any of the nonphosphorylated mutants of Traf6 decreased the total K63-linked polyubiquitination in whole-cell lysates, as detected with the antibody that specifically recognize K63-linked polyubiquitination (Fig. 3*C*). These results indicate that the phosphorylation of Traf6 by c-Src regulates its activity for K63-linked polyubiquitination. Meanwhile, mAEP levels were decreased after overexpressing these nonphosphorylated mutants of Traf6 with c-Src (Fig. 3*C*). Among the phosphorylated sites in Traf6, blocking the phosphorylation of Traf6 at Y473 induced the most significant decrease in mAEP levels (Fig. 3, *C* and *D*). We made a pY473-Traf6 antibody and confirmed the phosphorylation of Traf6 at Y473 by c-Src after an *in vitro* kinase reaction by Western blotting and a dot blot assay (Fig. 3*E*). Furthermore, we found that EGF treatment also induced the phosphorylation of Traf6 at Y473 in parallel with AEP activation, which was inhibited by WH-4-023 (Fig. 2, *A* and *B*). Overall, these results suggest that c-Src regulates mAEP levels by phosphorylating Traf6.

### Inhibition of Src reduces Traf6 phosphorylation and decreases mAEP in neurons derived from hiPSCs

**Figure 4:**
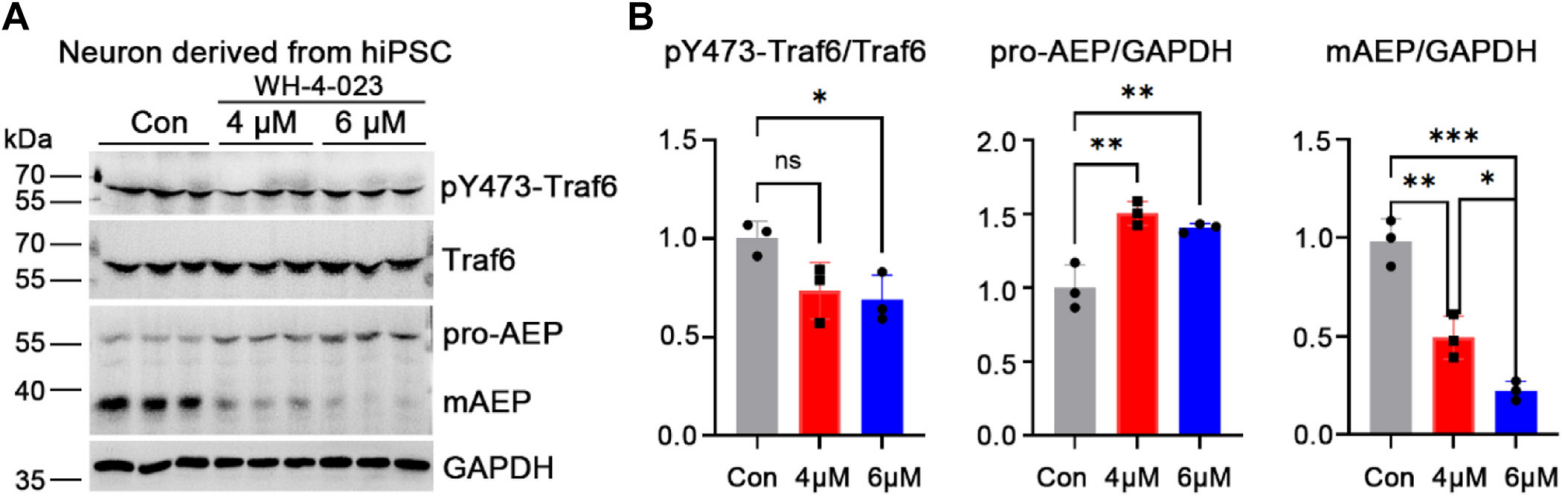
**Inhibition of Src reduces phosphorylation of Traf6 and inactivates AEP in neurons derived from human-induced pluripotent stem cells (hiPSCs).***A*, Western blot showing Traf6 and AEP levels in hiPSC-derived neurons treated with WH-4-023. *B*, quantification of the ratios of the gray values of pY473-Traf6/Traf6, pro-AEP/GAPDH, and mAEP/GAPDH. Data are presented as the mean ± SD, n = 3 for each group. ∗*p* < 0.05, ∗∗*p* < 0.01, ∗∗∗*p* < 0.001, ns indicates nonsignificant. AEP, asparaginyl endopeptidase; mAEP, mature AEP; Traf6, tumor necrosis factor receptor–associated factor 6.

The aforementioned results clearly showed that the effect of Traf6 on AEP activation was regulated by c-Src in HEK-293T cells. However, whether the c-Src–Traf6–AEP pathway exists in neurons is still unknown. To confirm this hypothesis, we treated neurons derived from hiPSCs (Fig. 3, *A*–*C*) with the Src inhibitor WH-4-023. After 48 h of treatment, the Src inhibitor WH-4-023 inhibited the phosphorylation of Traf6 at Y473 (Fig. 4, *A* and *B*). Intriguingly, it inhibited AEP activation in a dose-dependent manner (Fig. 4, *A* and *B*), suggesting that c-Src phosphorylates Traf6 under physiological conditions and that the inhibition of Src decreases the phosphorylation of Traf6 and inhibits AEP activation in neurons derived from hiPSCs.

### Traf6 mediates c-Src-induced AEP-cleaved Tau production *in vivo*

**Figure 5:**
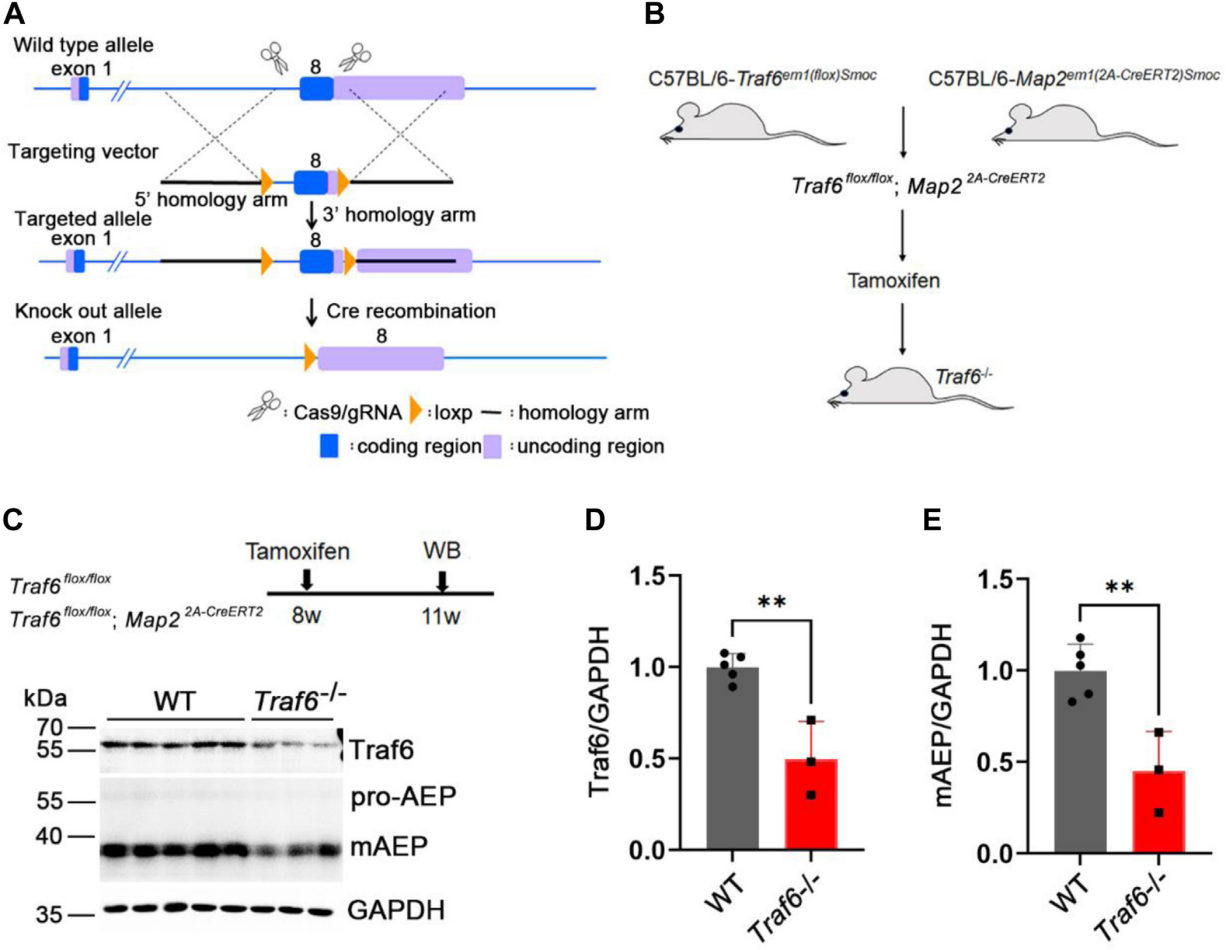
**Neuronal Traf6 regulates mAEP levels *in vivo*.***A*, schematic representation of the strategy for constructing conditional Traf6 knockout mice. *B*, diagram illustrating the conditional knockout of Traf6 specifically in neurons. *C*, Western blot showing that knockout of Traf6 (Traf6^−/−^) decreased mAEP levels in the hippocampus. *D* and *E*, quantification of the ratio of Traf6 and mAEP to GAPDH in (*C*). WT, n = 5; Traf6^−/−^, n = 3. Data are presented as the mean ± SD. ∗∗*p* < 0.01. mAEP, mature asparaginyl endopeptidase; Traf6, tumor necrosis factor receptor–associated factor 6.

To explore whether Traf6 mediates c-Src-induced AEP activation in neurons *in vivo*, we first performed conditional knockout of Traf6 in mature neurons in *Traf6*^flox/flox^*;MAP2*^Cre-ERT2^ (*Traf6*^−/−^) mice (Fig. S4*A*) by the IP injection of tamoxifen into the mice for a total of 5 consecutive days (Fig. 5, *A* and *B*). Three weeks after the injection, the Traf6 protein level had decreased by approximately 50% in the *Traf6*^−/−^ mice (Fig. 5, *C* and *D*). The remaining 50% of the Traf6 was probably expressed in glial cells or lost because of the insufficient deletion of *Traf6* in neurons. The loss of Traf6 in mature neurons resulted in a 50% reduction in the mAEP level in *Traf6*^−/−^ mice compared with WT mice (Fig. 5, *C* and *E*). These *in vivo* results indicate that neuronal Traf6 regulates mAEP levels.

**Figure 6:**
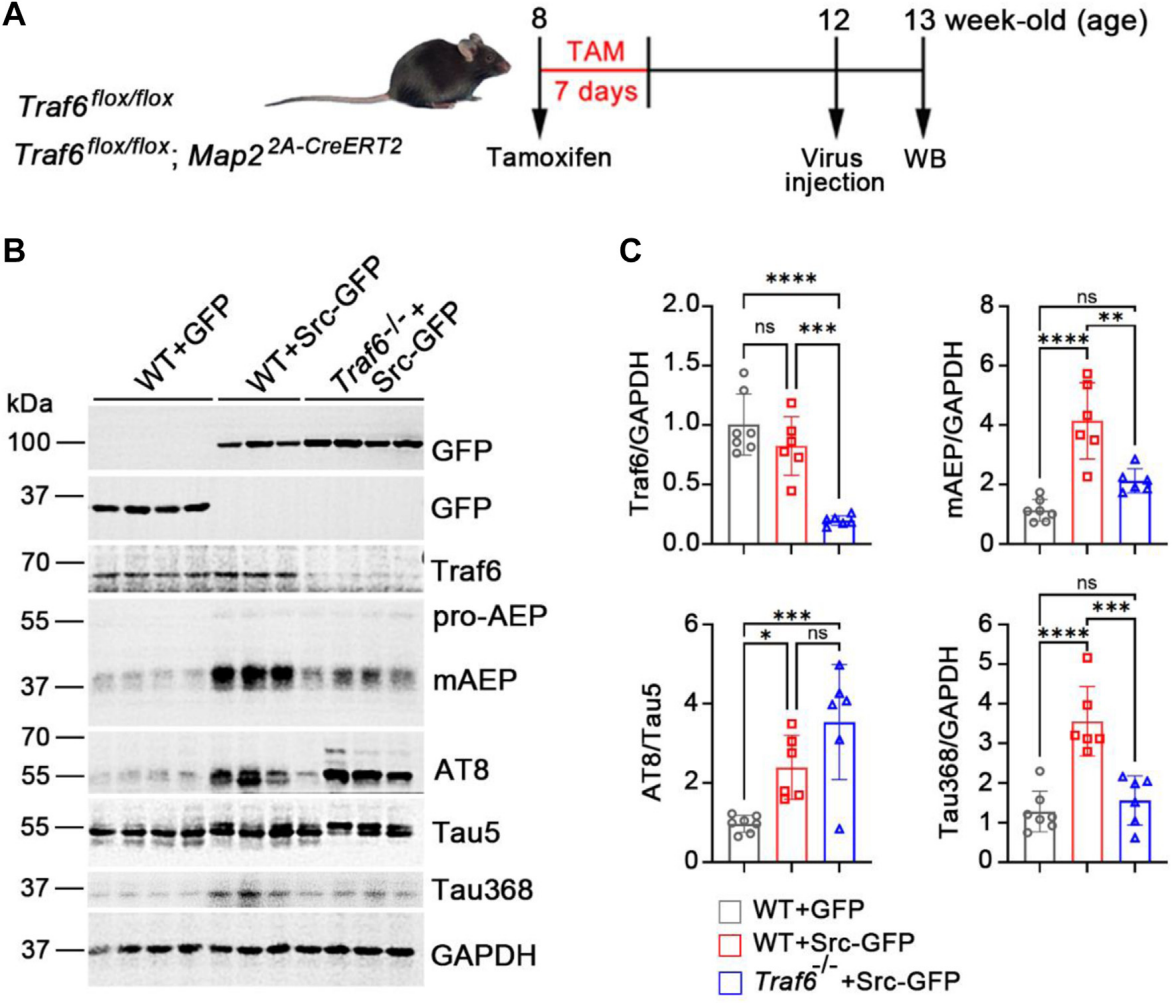
**c-Src induces Tau truncation and phosphorylation *in vivo*.***A*, schematic representation of the overexpression of activated c-Src in neuronal Traf6 knockout mice. Mice received intraperitoneal (IP) injection of tamoxifen at 8 weeks and injection of the virus into the CA1 region at 12 weeks. The hippocampus was isolated and processed for Western blotting 1 week after injection. *B*, Western blot showing c-Src-GFP, Traf6, AEP, AT8, Tau5, Tau368, and GAPDH using their specific antibodies. *C*, quantification of the ratio of Traf6, mAEP, Tau368 to GAPDH, and AT8 to Tau5 in (*B*). WT mice overexpressing GFP, n = 7; WT mice overexpressing Src-GFP, n = 6; Traf6^−/−^ mice overexpressing Src-GFP, n = 6. Data are presented as the mean ± SD. ∗*p* < 0.05, ∗∗*p* < 0.01, ∗∗∗*p* < 0.001, ∗∗∗∗*p* < 0.0001, ns, nonsignificant. mAEP, mature asparaginyl endopeptidase; Traf6, tumor necrosis factor receptor–associated factor 6.

After confirming that Traf6 regulates mAEP in neurons, we injected AAV2/8-hSyn-c-Src (Y535F)-enhanced GFP (EGFP)-3×FLAG-SV40 PolyA, a continuously activated form of c-Src (Y535F) with the hSyn promoter, or its control virus, AAV2/8-hSyn-EGFP-3×FLAG-SV40 PolyA, into the CA1 region of the hippocampus in WT and *Traf6*^−/−^ mice at 3 months of age, which received an IP injection of tamoxifen at 2 months of age for a total of 7 consecutive days to efficiently induce *Traf6* deletion in neurons (Fig. 6*A*). One week after virus injection, the hippocampus was isolated from the mice and processed for Western blotting. Blotting of the proteins with a GFP antibody revealed that the virus was expressed (control EGFP is 37 kDa, Src-EGFP is approximately 100 kDa) (Fig. 6*B*). The conditional KO of Traf6 was confirmed by immunoblotting with a Traf6 antibody, which showed an ∼80% decrease in the protein level in *Traf6*^−/−^ mice compared with WT mice (Fig. 6, *B* and *C*). Consistent with the results that we observed in HEK-293T cells, overexpressing c-Src in the hippocampus led to a dramatic increase in mAEP levels compared with those in the control group, whereas knockout of Traf6 suppressed the c-Src-induced increase in mAEP (Fig. 6, *B* and *C*). Parallel to the changes in mAEP levels, the level of AEP-cleaved Tau (Tau368) was increased in c-Src-overexpressing mice, whereas the knockout of Traf6 inhibited the c-Src-induced increase in Tau368 (Fig. 6, *B* and *C*). c-Src, as an upstream kinase of PP2Ac, phosphorylates and inactivates its activity, which leads to Tau phosphorylation (11). We also observed that Tau phosphorylation increased after c-Src overexpression, but it was not influenced by the deletion of Traf6 (Fig. 6, *B* and *C*). Altogether, these data suggest that the activation of c-Src promotes AEP-cleaved Tau production *via* Traf6 except that it induces Tau phosphorylation through PP2A *in vivo*.

### The activation of Traf6 is correlated with Tau pathology in control and AD brains

**Figure 7:**
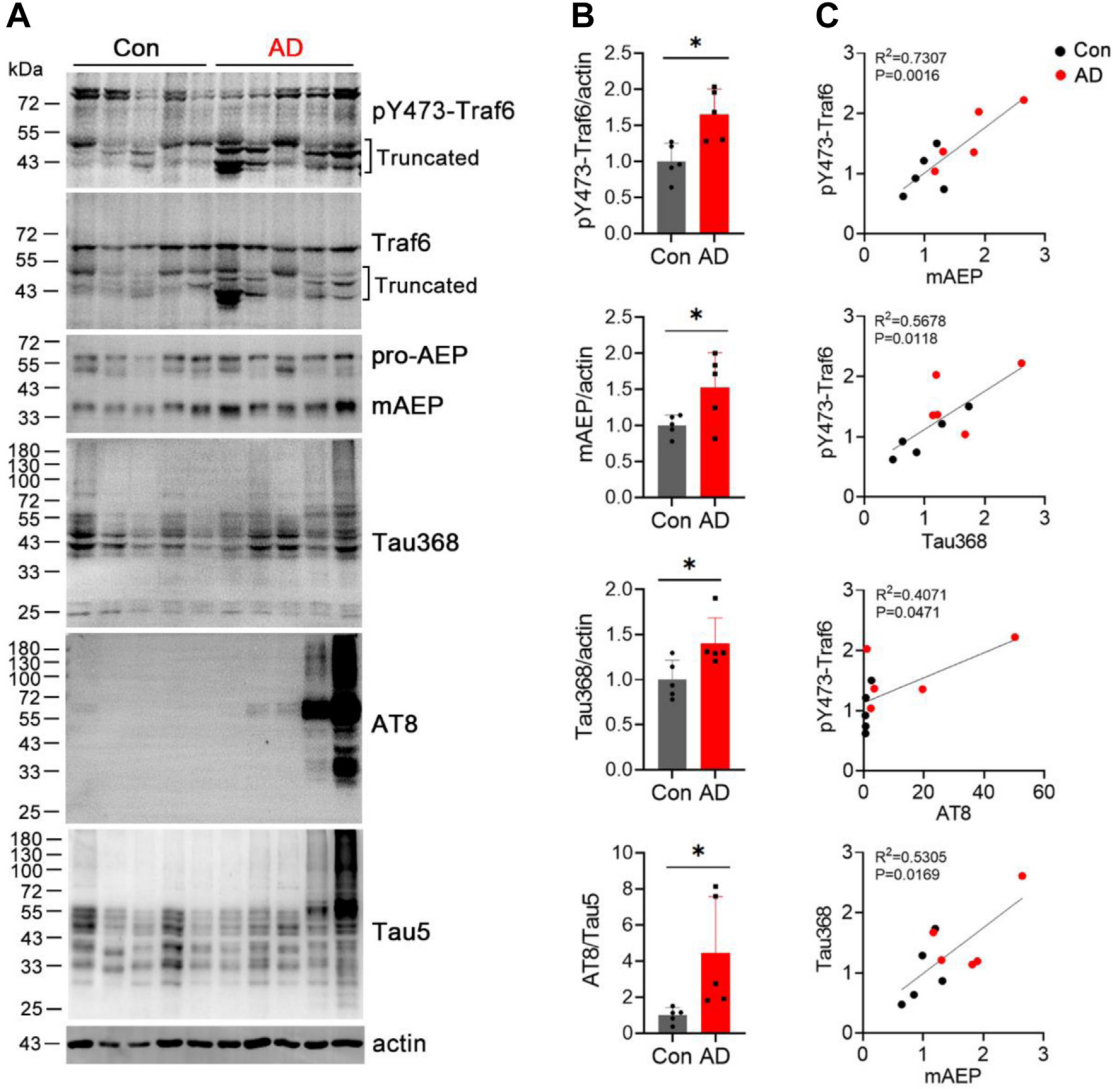
**The activation of Traf6 by c-Src is positively correlated with Tau pathology in AD brain.***A*, Western blotting of Traf6, pY473-Traf6, AEP, Tau368, AT8, Tau5, and actin in the brain of control and AD patients. *B*, quantification of the ratio of pY473-Traf6, mAEP, Tau368 to actin, and AT8 to Tau5 in (*A*). *C*, the linear correlation between pY473-Traf6 and mAEP, pY473-Traf6 and Tau368, pY473-Traf6 and AT8, and mAEP and Tau368 was analyzed. Con, n = 5; AD, n = 5. Data are presented as the mean ± SD. ∗*p* < 0.05. AD, Alzheimer’s disease; AEP, asparaginyl endopeptidase; Traf6, tumor necrosis factor receptor–associated factor 6.

In order to further prove that Traf6 is involved in the development of Tau pathology, we detected alterations of pY473-Traf6, Traf6, AEP, Tau368, AT8, and Tau5 in control and AD brains (Fig. 7*A*). Quantification of the blots indicated that the protein levels of pY473-Traf6, mAEP, Tau368, and AT8 were significantly increased in AD brains compared with the control brains (Fig. 7*B*). These results suggest that Traf6 and AEP are activated, and AEP-cleaved Tau368 and phosphorylation of Tau are increased in AD brains compared with the control brains.

To investigate the association between the phosphorylation of Traf6 by c-Src and Tau pathology, we performed linear correlation analysis and found that pY473-Traf6 protein level was positively correlated with mAEP, Tau368, and AT8 protein levels, respectively (Fig. 7*C*). These data suggest that the activation of Traf6 is correlated with Tau pathology in control and AD brains.

## Discussion

The activation of Src family kinases has been implicated in many neurodegenerative diseases. Fyn was shown to control Tau aggregation in an AD mouse model (13), whereas c-Abl phosphorylates parkin and inhibits its protective function in Parkinson’s disease (19). Furthermore, c-Src has been reported to mediate zinc-induced Tau phosphorylation (20). Notably, c-Src is enriched in neurons and plays a vital role in long-term potentiation and spatial learning (21, 22). In fact, inhibition of c-Src has been shown to protect against cerebral damage after stroke (23). Both c-Src and Fyn regulate Tau phosphorylation by inhibiting the phosphatase PP2A. These findings suggest that c-Src activation might contribute to AD pathology and disease progression.

In our study, we found that c-Src regulates the truncation of Tau by AEP. However, the mechanisms underlying changes in c-Src activity in AD require further investigation. Moreover, there are concerns about the long-term inhibition of c-Src to improve cognitive function in Tau mouse models, as c-Src is required for long-term potentiation in CA1 hippocampal neurons (21). Therefore, chronic inhibition of c-Src in AD mouse models should be carefully evaluated in future studies.

c-Src and Traf6 have been reported to interact and regulate each other (10). However, the precise mechanism by which c-Src regulates Traf6, an E3 ligase enzyme, remains unknown. Our MS analysis clearly showed that c-Src phosphorylates Traf6 at the sites Y326, Y381, Y353, Y473, Y68, and Y288, with a preference for Y326 (Fig. 3*B*). Surprisingly, any single mutation at these phosphorylation sites suppressed K63-linked polyubiquitination in the total-cell lysate from HEK-293T cells (Fig. 3*C*), which is consistent with the known function of Traf6 (24). The expression of nonphosphorylated mimic mutants also reduced AEP activation (Fig. 3*C*).

The human proteome comprises approximately 600 E3 ligases, which can be classified into two classes: those containing a HECT domain and those containing a RING or RING-like domain (25). Traf6 and RNF168 are two known E3 ligases that selectively target substrates for K63-linked ubiquitination (26). K63-linked ubiquitination of Traf6 plays a role in nondegraded signaling and has been implicated in endocytosis, autophagy, DNA damage impairment, innate immunity, and signaling conduction (25). Despite the ability of Traf6 to mediate AEP polyubiquitylation and promote pro-AEP stability by recruiting HSP90α (9), the polyubiquitylation of AEP and pro-AEP stability appear to be two closely related but separate events. This is supported by our results, which show that the molecular weights of pro-AEP and mAEP did not differ in Western blots (Fig. 2), consistent with previous findings (9). It is possible that the interaction of Traf6 and pro-AEP promotes pro-AEP stability by recruiting HSP90α, whereas the activity of Traf6 regulated by c-Src controls pro-AEP maturation. As shown in Figure 3, our findings indicate that nonphosphorylated mutations in Traf6 slightly increase pro-AEP levels but dramatically decrease mAEP levels. In addition, as shown in Figures 1*A* and 4, inhibition of c-Src by WH-4-023 only reduced mAEP levels while increasing pro-AEP levels. In contrast, knockdown or knockout of Traf6 *in vitro* and *in vivo* reduced pro-AEP and mAEP levels, as shown in Figures 2, 5 and 6. These findings suggest that Traf6 plays a role in regulating the stability of AEP, whereas its activation influences AEP activation. Unlike the suppression of enzyme activity, knockdown or knockout of Traf6 or c-Src could change their interacted proteins. One should carefully compare these results. However, the specific mechanism by which Traf6 activation affects AEP activation requires further exploration. In addition, the reduced phosphorylation of Traf6 and AEP activation observed in neurons derived from hiPSCs treated with a Src inhibitor further support the existence of a c-Src–Traf6–AEP activation axis (Fig. 4).

AEP is an enzyme found in both the lysosome and the cytosol (9). Interestingly, even when the lysosome was removed from the cytosol, we still observed the presence of mAEP in the cytosol (Fig. 1*H*). This localization of AEP in the cytosol is intriguing because it offers additional possibilities for the enzyme to cleave its substrates during the aging process. However, the specific physiological function of AEP in the cytosol remains unclear.

AEP cleaves the Tau protein at N167, N255, and N368 (27). Truncated Tau cleaved by AEP shows increased aggregation and neurotoxicity (8). Abnormal activation of AEP regulates both amyloid-β and Tau pathology in AD (28). Here, we further demonstrated the regulation of AEP by Traf6 in neurons in mice. This implies that the abnormal activation of Traf6 could promote AEP-cleaved Tau production in neurons. By using a Tau368 antibody that specifically recognizes truncated Tau produced by AEP, our results showed that Traf6 mediated c-Src-induced AEP activation, thereby promoting Tau truncation *in vivo* (Fig. 6). However, an increased cleavage of Tau was not accompanied by a decreased quantity of full-length Tau. This is consistent with changes of Tau and Tau368 in AD brain and PS19 mouse model. Tau stabilizes microtubules in its full-length form in physiological condition. Proteolytic Tau may account for only a fraction of total Tau, which is not enough to reduce full-length Tau level.

**Figure 8:**
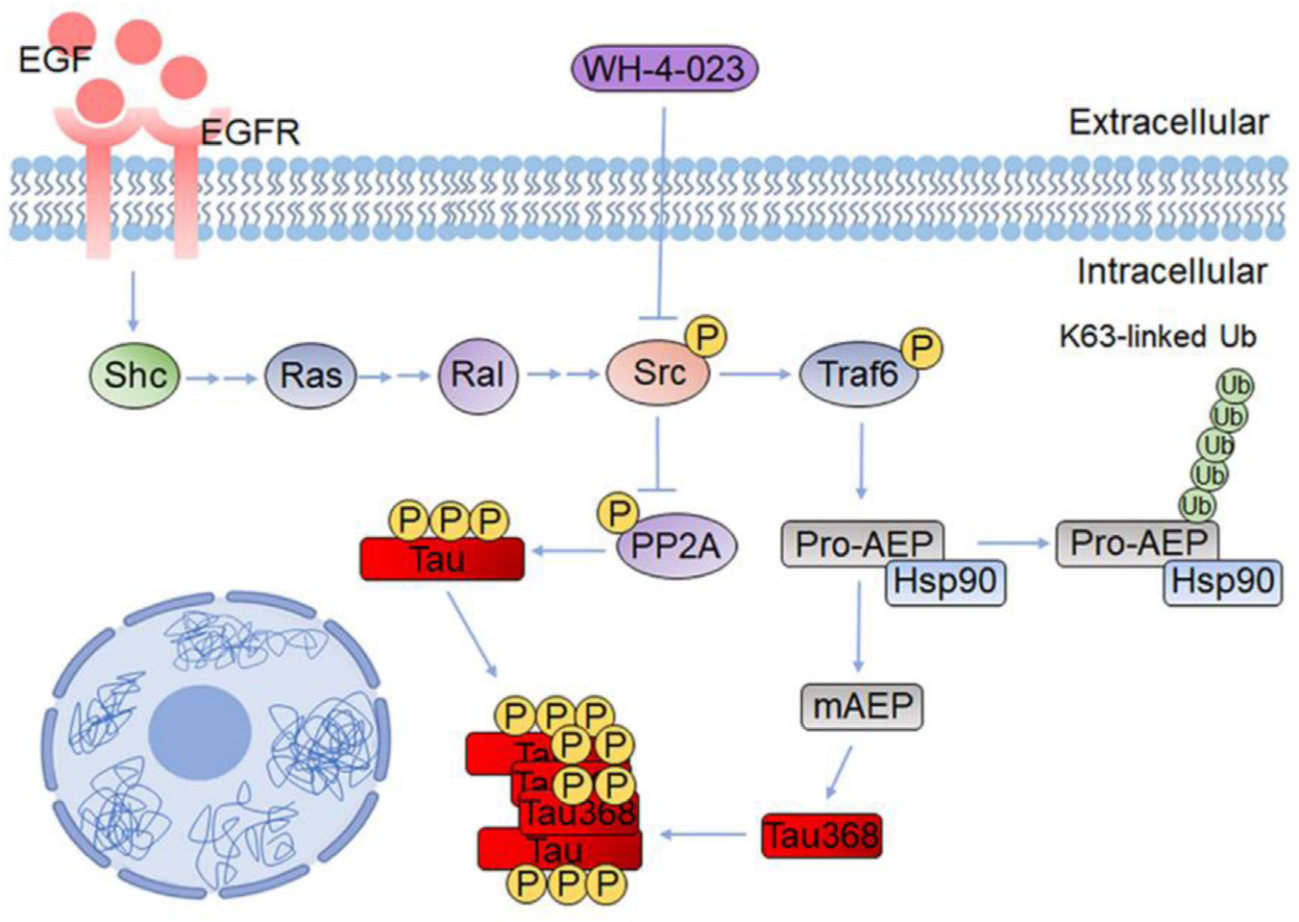
**Mechanism of Tau pathology induced by c-Src.** Schematic diagram showing c-Src–Traf6–AEP–Tau pathology pathway. Under EGF treatment, c-Src influences AEP activity by phosphorylating Traf6, promoting AEP-induced Tau truncation. Pharmacologic inhibition of c-Src with WH-4-023 in neurons derived from hiPSCs or genetic depletion of Traf6 in c-Src Y573F-overexpressed mice downregulates mAEP level and Tau truncation. Activation of c-Src can also inhibit PP2A activity and increase Tau phosphorylation. Phosphorylated and truncated Tau368 form aggregates and induce endogenous Tau pathology. AEP, asparaginyl endopeptidase; EGF, epidermal growth factor; hiPSC, human-induced pluripotent stem cell; mAEP, mature AEP; PP2A, protein phosphatase 2A; Traf6, tumor necrosis factor receptor–associated factor 6.

In conclusion, our findings provide evidence that c-Src plays a crucial role in regulating the production of AEP-cleaved Tau through the phosphorylation of Traf6. Phosphorylation of Traf6 is highly correlated with AEP activation, Tau truncated at N368, and pathologic Tau accumulation (AT8) in AD brain. Thus, the activation of Traf6 by c-Src appears to be associated with the development of Tau pathology in AD. Therefore, targeting the abnormal activity of c-Src could yield significant therapeutic benefits in AD treatment. By inhibiting abnormal c-Src activity, both Tau phosphorylation and truncation may be reduced, thereby alleviating the progression of AD pathology (Fig. 8).

## Experimental procedures

### Plasmids and antibodies

The plasmid encoding c-Src with a 3×FLAG tag was purchased from GeneChem, and Traf6 with specific site mutations and a 3×FLAG tag was synthesized and constructed in the pcDNA3.1 vector by Sangon Biotech Co, Ltd and prepared using an endotoxin-free plasmid extraction kit (Tiangen). The antibodies used in this study are listed in Table S1. The pY473-Traf6 polyclonal antibody was customed by Wuhan Bioyeargene Biotechnology Co, Ltd. Briefly, peptides of amino acids surrounding the phospho-Tyrosine 473 and non–phospho-Tyrosine 473 site of human Traf6 were synthesized (antigen). The structure and purity of these two peptides were confirmed by HPLC. Next, the keyhole limpet hemocyanin was conjugated to peptides through Sulfo-SMCC (immunogen). And the prepared immunogens were mixed and emulsified with the Freund’s adjuvant and injected subcutaneously to immunize rabbits four times within 45 days to efficiently produce antibody. Then, carotid blood was collected, and the antibody titer in the serum was detected by ELISA (these two peptides were used to coat ELISA plate). Once the antibody titer in the serum met the requirement, the serum was collected, and antibody was purified from serum by affinity chromatography. And the titer and specificity of purified antibody were detected by ELISA (phospho-Y473 peptide–coated ELISA plate was used to detect the purified pY473-Traf6 antibody, and the nonphospho-Y473 peptide–coated ELISA plate was used as negative control).

### Animal model

*Traf6*^flox/flox^ mice (Shanghai Model Organisms, NM-CKO-200144) were crossed with *Map2*^CreERT2/+^ mice (Shanghai Model Organisms, NM-KI-00037) to obtain *Traf6*^flox/flox^;*Map2*^CreERT2/+^ mice and littermate control *Traf6*^flox/flox^ mice. All the mice were on the C57BL/6 background. The mice were housed no more than five per cage in a pathogen-free barrier facility and given ad libitum access to food and water. Both male and female mice were used in this study. The animals were kept in the animal colonies with a 12 h light–dark cycle. All animal handling and use abided by a protocol approved by the Institutional Animal Care and Use Committee at the Tongji Medical School of the Huazhong University of Science and Technology.

### Tamoxifen administration

To induce efficient Cre expression and recombination *in vivo*, tamoxifen (T5648; Sigma–Aldrich) was dissolved in corn oil to prepare a 20 mg/ml stock. The mice were given tamoxifen at 75 mg/kg per day *via* IP injection for 5–7 days as indicated at 2 months of age.

### Cell culture

HEK-293T cell line was purchased from Wuhan Procell Life Technology Co, LTD. The authenticity of HEK-293T cells was validated using short tandem repeat profiling. This cell line was tested for mycoplasma and was free from mycoplasma contamination for all experiments. HEK-293T cells were cultured in Dulbecco’s modified Eagle’s medium (DMEM) supplemented with 10% fetal bovine serum (Gibco BRL), 100 U/ml penicillin, and 100 μg/ml streptomycin (Invitrogen) and incubated in a humidified atmosphere containing 5% CO_2_ at 37 °C. The cells were seeded on culture plates, and all transfection experiments were performed with Neofect (Neofect Biotech) according to the manufacturer’s instructions. Empty vectors were used for the corresponding controls.

### Treatment of cells with EGF

HEK-293T cells were starved overnight in medium without fetal bovine serum. The cells were preincubated with or without the Src inhibitor WH-4-023 (10 μM, MedChemExpress, HY-12299; WH-4-023 is a potent and selective dual Lck/Src inhibitor with IC_50_ of 2 nM/6 nM for Lck and Src kinase, respectively; little inhibition on p38α and KDR) for 30 min and treated with recombinant human EGF (Sangon Biotech, C610033) (200 ng/ml) for another 30 min. Then, the cells were lysed, and Western blotting was performed.

### Immunoprecipitation

Fifty microliters of Protein G or A Magnetic Beads (Biolinkedin) for each sample were washed three times with PBS and incubated with antibody (anti-Src, Millipore, 05-184; anti-AKT, CST, 4691; anti-AEP, R&D, AF2058, sheep immunoglobulin G) in PBS (1 μl per 100 μg of protein) for 4 h at 4 °C. The beads were isolated, washed three times with PBS, and incubated with cell lysate in radioimmunoprecipitation assay (RIPA) buffer (50 mM Tris [pH 7.4], 150 mM NaCl, 1% Triton X-100, 0.25% sodium deoxycholate, and 0.1% SDS) supplemented with protease inhibitor cocktail (Sigma‒Aldrich; P8340) overnight at 4 °C. The next day, the beads were isolated, washed three times with RIPA buffer, and boiled in 50 μl of 1× Laemmli SDS sample buffer for 5 min. The elution buffer was separated and used for SDS-PAGE.

### Isolation of the cytosolic fraction

Cells were washed with cold PBS and frozen/thawed once at −80 °C, and the cell lysate was suspended in buffer containing 0.25 M sucrose, 1 mM EDTA disodium, 10 mM Hepes, pH 7.4. The cell lysate was centrifuged at 750*g* for 10 min at 4 °C in a fixed angle rotor. The supernatant was kept and referred to as the total fraction. A part of the total fraction was centrifuged at 20,000*g* for 10 min at 4 °C in a fixed angle rotor, and then the supernatant was centrifuged at 105,000*g* for 1 h at 4 °C. The resulting supernatant was saved as the cytosolic fraction.

### RNA interference

HEK-293T cells were plated in 6-well plates, with each group in triplicate. Two microliters of Src shRNA (target: gcTCGGCTCATTGAAGACAAT) plasmid was mixed with 2 μl of Neofect DNA transfection reagent in Opti-MEM (Gibco), incubated for 15 min at room temperature (RT) and added to one well of 6-well plates. Forty-eight hours after transfection, the cells were lysed in RIPA buffer (50 mM Tris [pH 7.4], 150 mM NaCl, 1% Triton X-100, 1% sodium deoxycholate, and 0.1% SDS) with a protease inhibitor cocktail. The protein concentration was measured with a bicinchoninic acid (BCA) Protein Assay kit (Thermo Fisher Scientific; 23225) according to the manufacturer’s instructions, and the protein was stored at −20 °C.

For Traf6 3′UTR siRNA interference, 60 pM of siRNA was mixed with 3 μl of RNATransMate (Sangon Biotech; E607402) for 5 min in 100 μl of Opti-MEM and added to the cell medium directly for one well of 6-well plates. Forty-eight hours after transfection, the cells were treated as described previously. The siRNA sequences were as follows:

Negative control: sense 5′-3′: UUCUCCGAACGUGUCACGUTT; antisense 5′-3′: ACGUGACACGUUCGGAGAATT;

hTraf6: sense 5′-3′: GCCACGGGAAAUAUGUAAUAUTT; antisense 5′-3′: AUAUUACAUAUUUCCCGUGGCTT.

### c-Src phosphorylates Traf6 *in vitro*

c-Src tagged with 3×FLAG was overexpressed in HEK-293T cells in 10 cm plates for 48 h. Then, the cells were treated with EGF (200 ng/ml) for 30 min to stimulate c-Src, the cells were lysed in RIPA buffer, and c-Src was pulled down by using anti-FLAG Magnetic Beads (Biolinkedin). Magnetic beads bound with c-Src were mixed with 10 μg of recombinant human Traf6 protein (CUSABIO, CSB-EP024154HU, with 6×His-SUMO tag at N-terminal region) in 50 μl of kinase reaction buffer (50 mM Tris–HCl, pH 7.4, 10 mM MgCl_2_, 5 mM 1,4-DTT, and 1 mM ATP) in a water bath at 37 °C for 40 min. Then, the sample was quickly frozen in dry ice to stop the reaction.

### Western blotting

Mouse brain samples or cultured cells were lysed in RIPA buffer and added to 4× Laemmli SDS sample buffer at a ratio of 3:1, followed by boiling for 10 min. The protein concentration was measured by a BCA Protein Assay kit. Samples were subjected to SDS-PAGE and transferred onto a polyvinylidene fluoride membrane (MilliporeSigma). The membrane was subsequently blocked with 5% fat-free milk in TBS (Tris-buffered saline) for 30 min, incubated with primary antibody (Table S1) diluted in 5% fat-free milk in TBS at 4 °C overnight, washed three times with TBS with 0.1% Tween-20, incubated with horseradish peroxidase–conjugated secondary antibody for 2 h at RT, washed three times with TBS with 0.1% Tween-20, incubated with ECL Western blotting substrate (Thermo Fisher Scientific), and exposed to X-OMAT BT Film or ChemiScope (Clinx Science Instruments Co, Ltd). Specific immunostaining was quantified by using Multi Gauge software V3.0 from Fuji Film (Minato).

### Dot blotting

Dot blotting was performed as previously described with little modification (29). Two microliters of reaction buffer (0 and 40 min) containing Traf6 (0.167 μg/μl) was added to the grid of a nitrocellulose membrane and dried at 37 °C for 1 h. Each sample was prepared in triplicate. The membrane was blocked in 3% BSA in TBS for 30 min and incubated with primary antibody overnight. Then, the membrane was processed as described previously for Western blotting.

### Coomassie brilliant blue staining

After SDS-PAGE, the gel was incubated in Coomassie brilliant blue reaction buffer (0.1% w/v R-250, 25% isopropanol, and 10% glacial acetic acid) for 30 min at RT. Then, it was washed in destaining solution (30% methanol, 10% glacial acetic acid, and 60% deionized water) until the band was clear. The specific band was cut out for MS.

### MS

#### Sample preparation

Protein lanes were cut into pieces of approximately 1 mm^3^ and further destained with 15 mM K_3_[Fe(CN)_6_]/50 mM Na_2_S_2_O_3_ before the pieces became colorless. The gel pieces were then washed with distilled water followed by 50% acetonitrile (ACN)/100 mM NH_4_HCO_3_ (pH 8.0) three times and then incubated with 100% ACN. After incubation, the samples were reduced with 10 mM DTT at 65 °C for 1 h, followed by alkylation with 55 mM iodoacetamide at RT in the dark for 30 min. Finally, the gel pieces were washed with 100% ACN and dried in a vacuum concentrator. Overnight tryptic digestion was conducted in 50 mM NH_4_HCO_3_ at 37 °C. After in-gel digestion, the peptides were extracted with 60% ACN/5% formic acid aided by an ultrasonic bath. The collected peptide samples were vacuum dried and purified using C18 desalting columns. The eluate was vacuum dried and stored at −20 °C for later use.

#### LC‒MS/MS detection

LC‒MS/MS data acquisition was carried out on a timsTOF Pro mass spectrometer (BRUKER). Peptides were first loaded onto a C18 trap column (75 μm × 2 cm, 3 μm particle size, 100 Å pore size; Thermo) and then separated in a C18 analytical column (75 μm × 250 mm, 3 μm particle size, 100 Å pore size; Thermo). Mobile phase A (H_2_O, 0.1% formic acid) and mobile phase B (ACN, 0.1% formic acid) were used to establish the separating gradient. A constant flow rate set at 300 nl/min was used. For incremental dynamic mode analysis, the tims function was operated, and parallel accumulation–serial fragmentation scan mode was applied: each scan cycle's total time was 1.11 s, which consisted of an MS1 scan time of 0.1 s and MS2 scan time from the beginning of the rest time.

#### Data analysis

Raw MS data were analyzed with MaxQuant (version 2.0.1, Max-Planck-Institute of Biochemistry) using the Andromeda database search algorithm. Spectra files were searched against the UniProt human proteome database using the following parameters: label-free quantitation mode was checked for quantification; variable modifications, dimethyl (R), methyl (R), oxidation (M) & acetyl (protein N-term); fixed modifications, carbamidomethyl (C); digestion, trypsin/P; match between runs was used for identification transfer. The search results were filtered with a 1% false discovery rate at both the protein and peptide levels.

### hiPSC culture and passage

iPSCs derived from normal human skin fibroblasts were used in this study. The hiPSC lines were maintained on vitronectin (Gibco; A14700)-coated culture plates in complete Essential 8 medium (E8+, Gibco, A1517001). The medium was changed daily, and the cells were passaged every 5 to 6 days using 0.25% trypsin–EDTA (Gibco; 25200114) according to the manufacturer’s instructions.

### Neural progenitor cell induction from hiPSCs

Neural progenitor cell (NPCs) were generated from hiPSCs by dual inhibition of the SMAD signaling pathway using DMH1 and SB431542. After three passages, hiPSCs were digested with dispase (STEMCELL; 07913) and cultured in suspension in NPC induction medium (1:1 v/v mixture of DMEM/F12 [Gibco; 11320033] and neurobasal medium [Gibco; 21103049), 1× N-2 supplement [Gibco; 17502048], 1× B-27 supplement [Gibco; 17504044], 1× nonessential amino acids [NEAAs; Gibco; 11140050], 1× GlutaMax [Gibco; 35050061], 1× penicillin–streptomycin [Gibco; 15140163], 100 μM β-mercaptoethanol [Sigma‒Aldrich; 60-24-2], and 5 ng/ml recombinant human basic fibroblast growth factor [MedChemExpress; HY-P7331]), which was supplemented with 2 μM DMH1 (TOCRIS; 4126) and 2 μM SB431542 (TOCRIS; 1614). The medium was changed daily. On day 10, the formed embryonic bodies were plated on vitronectin-coated plates in NPC induction medium supplemented with 2 μM DMH1 and 2 μM SB431542. The medium was changed daily. Neural rosettes had formed after 6 days, and then the neural rosettes were picked manually and expanded in suspension in neural maintenance medium (1:1 v/v mixture of DMEM/F12 and neurobasal medium, 1× N-2 supplement, 1× B-27 supplement, 1× NEAAs, 1× GlutaMax, 1× penicillin–streptomycin, 10 ng/ml recombinant human basic fibroblast growth factor, and 10 ng/ml recombinant human EGF), forming neurospheres. After 3 weeks, the neurospheres were cut into 30 μm-thick slices and stained with SOX2 and Nestin antibodies to detect the efficiency of NPC induction from hiPSCs by immunofluorescence.

### Neural differentiation of NPCs

To obtain human neurons, neurospheres were plated on PLO (Sigma‒Aldrich)/Matrigel (Corning; 356234)-coated dishes and cultured in neural differentiation medium (1:1 v/v mixture of DMEM/F12 and neurobasal medium, 1× N2 supplement, 1× B27 supplement, 1× NEAAs, 1× GlutaMax, and 1× penicillin/–streptomycin) supplemented with 0.2 mM ascorbic acid (MedChemExpress; HY-14649) and 25 μM β-mercaptoethanol. The medium was changed daily. The cells were passaged every 6 to 7 days using Accutase (Gibco; A1110501) and cultured for approximately 7 weeks. Then, the efficiency of the neural differentiation of NPCs was identified by immunofluorescent staining with β3-tubulin and NeuN antibodies.

### Immunofluorescence

Cells on coverslips were fixed with 4% paraformaldehyde for 15 min, washed with PBS, and incubated with 0.3% Triton X-100 in PBS for 15 min at RT. After blocking with 5% normal goat serum, 0.1% Triton X-100, and 0.05% Tween-20 in PBS for 30 min, the cells were incubated with primary antibody (Table S1) in blocking solution overnight at 4 °C, washed with PBS, and incubated with Alexa 488/594/647-conjugated secondary antibody for 2 h at RT. After washing with PBS, the sections were then dropped with antifade mounting medium with 4′,6-diamidino-2-phenylindole (Beyotime Biotechnology) and set under a coverslip. Images were taken on a Zeiss LSM 800 confocal microscope.

### Treatment of neurons derived from hiPSCs with the Src inhibitor WH-4-023

NPCs were differentiated into neurons for up to 44 days, and then the neurons were treated with the Src inhibitor WH-4-023 at 0, 4, and 6 μM for 48 h and lysed in RIPA buffer. The protein concentration was measured by the BCA method. Then, the samples were diluted in 4× Laemmli SDS sample buffer, boiled for 10 min, and subjected to Western blotting.

### Adeno-associated virus injection

Briefly, mice were deeply anesthetized and placed in a stereotaxic frame. After craniotomy, a 1 mm diameter hole was made with a motorized minidrill, and the AAV2/8-hSyn-Src (Y535F)-EGFP-3×FLAG-SV40 PolyA virus and its control virus AAV2/8-hSyn-EGFP-3×FLAG-SV40 PolyA (titers: 3.03 × 10^13^ virus particles/ml, 2.5 μl) were unilaterally injected into the hippocampal CA1 region (−2.0 mm anterior/posterior, 1.5 mm medial/lateral to bregma, and −1.5 mm dorsal/ventral to the dura surface) by using a 10 μl Hamilton syringe custom made with a 30-gauge/0.5 inch/hypodermic needle (Hamilton Syringe Co) at a rate of 0.5 μl/min for a total time of 5 min. The needle was kept in position for another 3 min and then withdrawn slowly to prevent leakage of the liquid. The skin was sutured after injection, and the mice were allowed to completely recover on a soft warming pad before they were returned to their home cages. All animal handling and use abided by a protocol approved by the Institutional Animal Care and Use Committee at the Tongji Medical School of the Huazhong University of Science and Technology.

### Human brain tissue

Frozen hippocampus from autopsied and histopathologically confirmed AD and age-matched normal human brains were obtained from the China Brain Bank of Zhejiang University School of Medicine. This study was approved by the Medical Ethics Committee of Tongji Medical College (S162), Huazhong University of Science and Technology and was conducted using guidelines established in accordance with the Declaration of Helsinki principles. The detailed information is in Table S2.

### Statistical analysis

Data were subjected to normality and lognormality tests before comparison. One-way or two-way ANOVA followed by Tukey’s multiple comparisons test was used in this study. Comparisons between two groups were analyzed by unpaired two-tailed *t* test. For analysis of the correlation, Pearson's (data with normal distribution) correlation coefficient was calculated. Data are presented as the mean ± SD as indicated and were analyzed using GraphPad Prism 8 (GraphPad Software, Inc). Differences for which *p* < 0.05 were considered statistically significant.

## Data availability

The datasets used and/or analyzed during the current study are available from the corresponding author on reasonable request.

## Supporting information

This article contains supporting information.

## Conflict of interest

The authors declare that they have no conflicts of interest with the contents of this article.
