# Supplementary material for: c-Src regulates δ-secretase activation and truncated Tau production by phosphorylating the E3 ligase Traf6
Source: J Biol Chem. 2023 Nov 15;299(12):105462. doi: 10.1016/j.jbc.2023.105462 (PMC10711223; doi:10.1016/j.jbc.2023.105462)
Supplement: Supporting Figures S1–S4 and Tables S1 and S2 [file mmc1.pdf]

Supporting Information

c-Src regulates δ-secretase activation and truncated Tau production by phosphorylating the E3 ligase Traf6

Running title: c-Src regulates Tau truncation and phosphorylation

Yanli Jiang[1]*, Longfei Li[1]*, Ruozhen Wu[1], Liulin Wu[1], Bin Zhang[1], Jian-Zhi Wang[1,2], Rong Liu[1], Fei Liu[3†], Jing Wang[4†], Xiaochuan Wang[1,2,5†]

[1]Department of Pathophysiology, School of Basic Medicine, Key Laboratory of Education Ministry/Hubei Province of China for Neurological Disorders, Tongji Medical College, Huazhong University of Science and Technology, Wuhan 430030, China
[2]Co-innovation Center of Neuroregeneration, Nantong University, Nantong, JS 226001, China
[3]Department of Neurochemistry, Inge Grundke-Iqbal Research Floor, New York State Institute for Basic Research in Developmental Disabilities, Staten Island, NY 10314, USA
[4]Department of Immunology School of Basic Medicine, Tongji Medical College, Huazhong University of Science and Technology, Wuhan 430030, China
[5]Shenzhen Huazhong University of Science and Technology Research Institute, Shenzhen 518000, China

* These authors contributed equally to this paper
[†] Correspondence to:
Xiaochuan Wang, Ph.D., Professor, Department of Pathophysiology, School of Basic Medicine, Tongji Medical College, Huazhong University of Science and Technology, Wuhan 430030, China.
Email: wangxiaochuan@hust.edu.cn

Jing Wang, Ph.D., Professor, Department of Immunology School of Basic Medicine, Tongji Medical College, Huazhong University of Science and Technology, Wuhan 430030, China.
Email: wangjhxh@163.com

Fei Liu, Ph.D., Professor, Department of Neurochemistry, Inge Grundke-Iqbal Research Floor, New York State Institute for Basic Research in Developmental Disabilities, Staten Island, NY 10314, USA
Email: fei.liu@opwdd.ny.gov

This file includes:

Supplementary Figures S1 to S4; Supplementary Table S1 and Table S2

Supplementary Figures

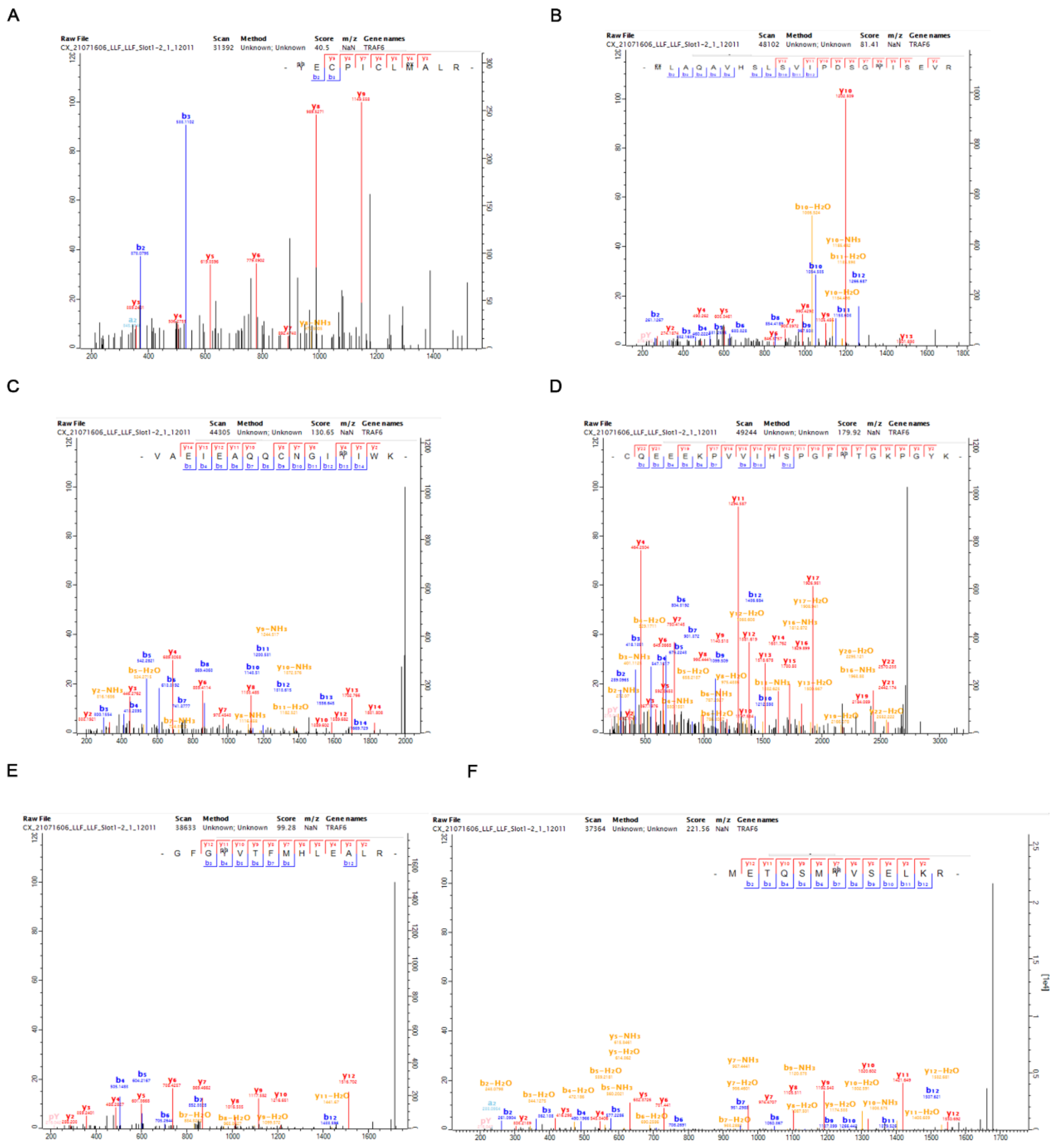

Supplementary Figure S1. Mass spectrum identification of sites in Traf6 that are phosphorylated by c-Src. (A) The spectrum of phosphorylated Traf6-Y68 [YECPICLMALR]. (B) The spectrum of phosphorylated Traf6-Y288 [MLAQAVHSLSVIPDSGYISEVR]. (C) The spectrum of phosphorylated Traf6-Y353 [VAEIEAQQCNGIYIWK]. (D) The spectrum of phosphorylated Traf6-Y381 [CQEEEKPVVIHSPGFYTGKPGYK]. (E) The spectrum of phosphorylated Traf6-Y473 [GFGYVTFMHLEALR]. (F) The spectrum of phosphorylated Traf6-Y326 [METQSMYVSELKR].

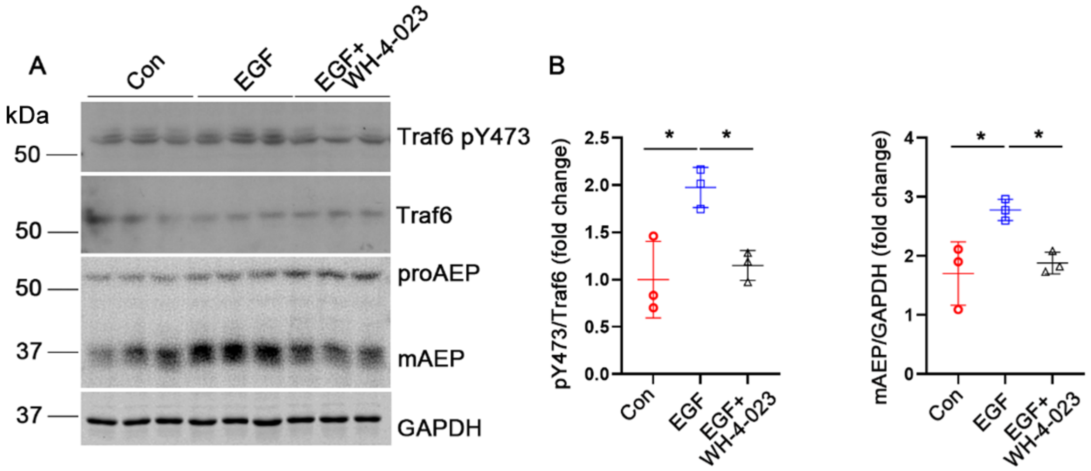

Supplementary Figure S2. c-Src mediates EGF-induced Traf6 phosphorylation and AEP activation. (A) HEK-293T cells were treated with EGF with or without WH-4-023 and processed for Western blot analysis. Membranes were blotted for Traf6, AEP, GAPDH, and pY473-Traf6 antibodies. (B) The ratios of pY473-Traf6 to Traf6 and mAEP to GAPDH, normalized to the control, were quantified. n=3 for each group, *p<0.05.

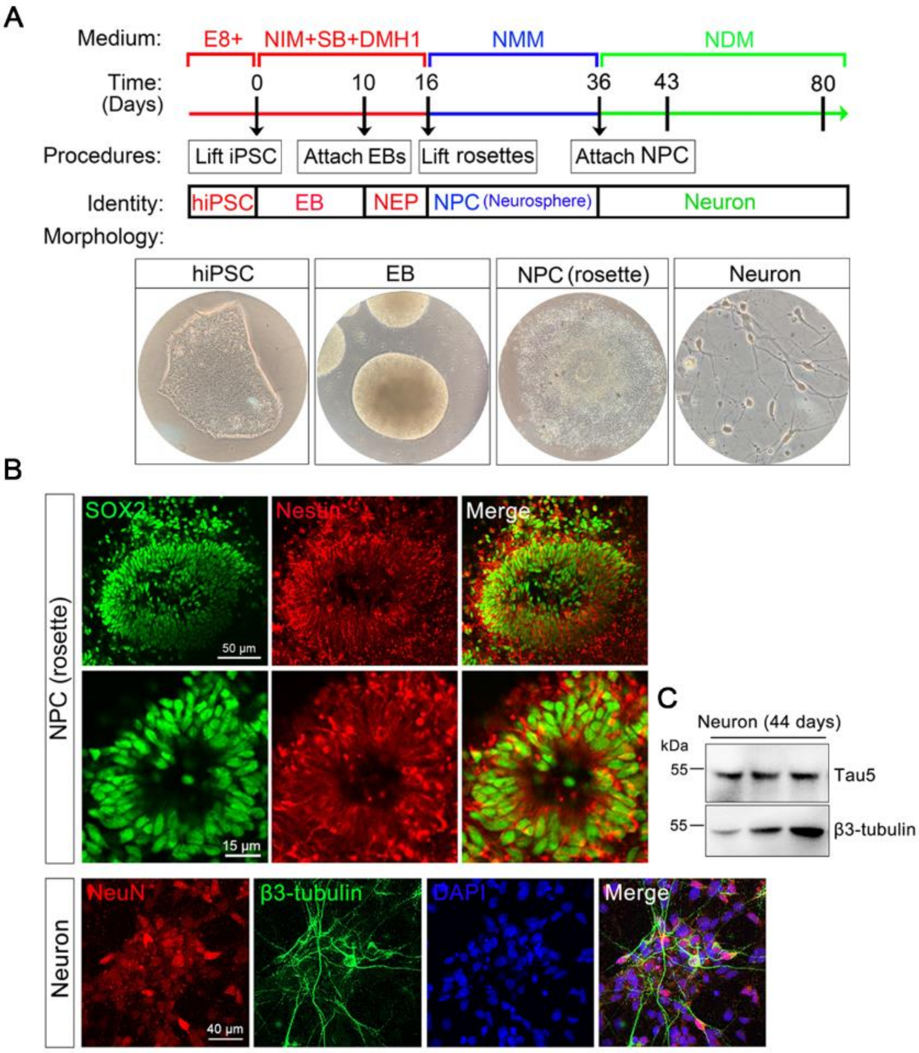

Supplementary Figure S3. Neuron derived from hiPSCs. (A) Schematic protocol for the differentiation of neurons from hiPSCs and morphology of cells during the induction process. The detailed procedure is described in the experimental procedures. Abbreviations: $E8^{+}$, Essential 8^{TM} medium; NIM, neural progenitor cell induction medium; NMM, neural maintenance medium; NDM, neural differentiation medium; EB, embryonic bodies; NPC, neural progenitor cell. (B) Immunofluorescent images showing the presence of SOX2- and Nestin-positive neuronal progenitor cell (NPC) (rosette formation) and immunofluorescence staining of differentiated neurons from hiPSCs using NeuN and β3-tubulin antibodies. (C) Western blot analysis of neurons derived from hiPSCs, detected using Tau5 and β3-tubulin antibodies.

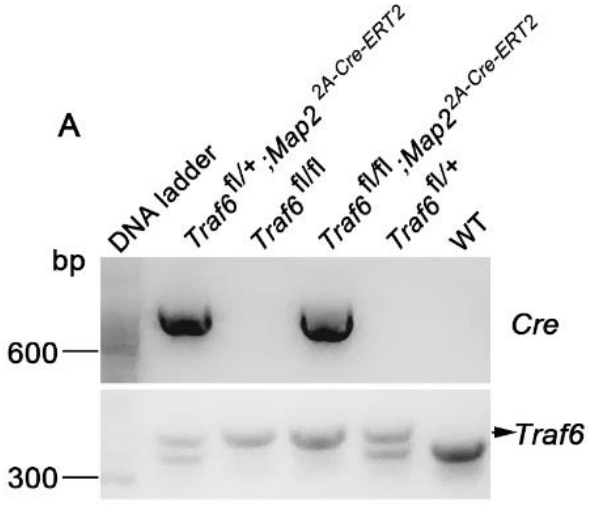

Supplementary Figure S4. Genotyping of the transgenic mice. (A) The upper panel shows the genotyping results, indicating the presence or absence of the Cre gene (700 bp band). (B) The lower panel displays genotyping results for wild-type mice (single band at 370 bp), Traf6$^{fl/+}$ mice (two bands at 370 bp and 428 bp), and Traf6$^{fl/fl}$ mice (single band at 428 bp).

Supplementary Table S1. Antibodies used in this study

Antibody	Type	Specificity	Species	Source/reference (cat/lot)
Tau5	Mono-	Tau (a.a.210-230)	M	Millipore (MAB361)
NeuN	Mono-	NeuN	R	CST (24307)
GAPDH	Poly-	GAPDH	R	Beyotime (AG019)
β-actin	Mono-	β-actin	M	Santa-Cruz (sc-25778)
Traf6	Poly-	Traf6	R	Zen bio (380803)
pY473-Traf6	Poly-	p-Traf6 (Y473)	R	Homemade
Src	Mono-	Src	M	Millipore (05-184)
Tau368	Poly-	Tau368	R	Millipore (ABN1703)
AEP	Poly-	AEP	R	CST (93627S)
SOX2	Poly-	SOX2	R	CST (3579)
Nestin	Poly-	Nestin	M	CST (33475)
β3-tubulin	Mono-	β3-tubulin	M	CST (4466)
AKT	Poly-	AKT	R	CST (9272)
pS473-AKT	Poly-	p-AKT (S473)	R	CST (4058)
GSK-3β	Mono-	GSK-3β	M	Abcam (ab9336)
pS9-GSK-3β	Poly-	p-GSK-3β (S9)	R	CST (14630)
Lamp1	Mono-	Lamp1	M	Santa-Cruz (sc-20011)
Ubi	Mono-	Ubiquitin	M	Abcam (ab7254)
AT8	Mono-	p-Tau (S202/T205)	M	Thermo (MN1020)
GFP	Mono-	GFP	M	Thermo (C163)
K63-Ubi	Poly-	K63	R	SIGMA (05-1308)
AEP	Poly-	AEP	Sh	R&D (AF2058)
Flag	Mono-	Flag	M	Millipore (MAB3118)

Abbreviations: Mono-, monoclonal; p-, phosphorylated; Poly-, polyclonal; M, mouse; R, rabbit; Sh, sheep.

Supplementary Table S2. Information of human samples

Case	Age	Sex	Brain region	Braak stage
Control-1	74	Male	Hippocampus	N/A
Control-2	83	Female	Hippocampus	N/A
Control-3	86	Male	Hippocampus	N/A
Control-4	98	Male	Hippocampus	N/A
Control-5	70	Female	Hippocampus	N/A
AD-1	83	Male	Hippocampus	4
AD-2	78	Male	Hippocampus	4
AD-3	71	Female	Hippocampus	4
AD-4	73	Male	Hippocampus	4
AD-5	79	Male	Hippocampus	6
